# ‘Whose role is it anyway?’ Experiences of community nurses in the delivery and support of oral health care for older people living at home: a grounded theory study

**DOI:** 10.1186/s12912-023-01533-0

**Published:** 2023-10-05

**Authors:** Gary Mitchell, Patrick Stark, Christine Brown Wilson, Georgios Tsakos, Paul Brocklehurst, Caroline Lappin, Barry Quinn, Nicola Holland, Gerry McKenna

**Affiliations:** 1 School of Nursing and Midwifery, Queen’s University Belfast, Belfast, Northern Ireland; 2 Department of Epidemiology and Public Health, University College London, London, UK; 3 School of Health Sciences, Bangor University, Bangor, UK; 4 Department of Health, Castle Buildings, Stormont, Belfast, Northern Ireland; 5 School of Medicine, Dentistry and Biomedical Sciences, Queen’s University Belfast, Belfast, Northern Ireland

**Keywords:** Oral health, Oral healthcare, Older people, Nursing, Community nursing, District nursing, Home healthcare, Home health care services, Grounded theory, Experiences

## Abstract

**Background:**

Older people who receive care at home are likely to require support with oral health care. Community nurses, who are also referred to as district or home care nurses, have an important role with this population. This is because they are the healthcare professionals who are most likely to encounter this population, who may also not be receiving regular dental care or oral health promotion. However, few studies have explored community nursing experiences in the delivery and support of oral healthcare for older people living at home.

**Methods:**

A grounded theory approach was used to explore experiences of community nurses in the delivery and support of oral health care for older people living at home. Fifteen practising community nurses from the United Kingdom participated in one-to-one semi-structed interviews from May 2021 to December 2021. These interviews were audio-recorded, transcribed verbatim and analysed using constant comparative analysis. Ethical approval was obtained for this study prior to data collection.

**Results:**

Four categories emerged from the data to support development of the core phenomena. These four categories were: (1) Education, in relation to what community nurses knew about oral health, (2) Practice, with regards to how community nurses delivered oral health care to older people in their own home, (3) Confidence, with consideration to the extent to which this supported or impeded community nurses in providing oral healthcare to older people and (4) Motivation, in terms of the extent to which community nurses thought they could or should influence future practice improvement in the area. The core category was (C) Uncertainty as it was both present and central across all four categories and related to community nursing understanding about their specific role, and the role of other professionals, with reference to oral health of their patients.

**Conclusions:**

This study reveals community nurses' uncertainty in providing oral healthcare to older adults at home. Emphasising comprehensive and continuous oral health education can boost nurses' confidence in patient support. Interprofessional collaboration and clear role definitions with oral health professionals are crucial for improving oral health outcomes in this vulnerable population.

**Supplementary Information:**

The online version contains supplementary material available at 10.1186/s12912-023-01533-0.

## Background

Healthy ageing has broadly been defined as the process of developing and maintaining functional ability that enables wellbeing in older age [1]. Functional ability includes many facets and relates to concepts like decision-making, mobility, nutrition, relationships, meaningful activity and participation in society [2–5]. Oral health is one of the key constituents of healthy ageing but is an area of practice and research that is often under prioritised [6–10]. With increasing age, a person is more likely to experience a decline in their health, have reduced functional ability and require higher dependence on informal or formal caregivers [11–13]. These factors make it more likely that an older person will require assistance to maintain good oral health [14–17].

It has been long established that an older person’s overall health can be predicted and measured by their oral health [18]. A person’s mouth, teeth, lips and tongue are important parts of the body and are essential for human functioning [19]. Across England, Wales, and Northern Ireland at least 1.8 million people aged 65 and over could have an urgent dental condition such as dental pain, oral sepsis, or extensive decay in untreated teeth [20]. There is also a plethora of evidence that suggests that older people, living at home, struggle to access dental services [20]. The needs for oral care are highest among those older people who live at home with complex comorbidities like dementia, heart failure, COPD, frailty or diabetes and do not regularly visit their dentist [1, 21].

Most oral health conditions experienced by older people are preventable or treatable but may remain undiagnosed due to poor understanding amongst healthcare staff, limited resources, inadequate policy protocols and sub-optimal multidisciplinary team working [22–26]. Despite this, oral health in Europe has improved in the last two decades and this has led to an increasing number of people retaining their natural teeth throughout their life [27]. However, this has also resulted in high levels of more complex oral health care needs as most older people have their natural teeth that are also heavily restored. And there are clear inequalities in oral health, with the more deprived population groups having considerably fewer natural teeth and higher levels of disease, both of which impair their function, compared to more affluent groups [28, 29]. While there is an increasing amount of research and evidence synthesis examining the effectiveness of oral health community-based interventions for older people living in long-term care settings, there has been a surprising paucity of research focused on older people receiving care at home [30–36].

Over the past two decades, the provision of healthcare for older people at home has increased significantly across Europe and the rest of the world [37]. Home care nursing, or community nursing as it is referred to in the United Kingdom, is a crucial service for many older people living at home [38, 39]. This is because community nurses will provide care to older people living at home with complex medical conditions such as frailty, dementia, heart failure or chronic obstructive pulmonary disease [40]. Community nurses will also provide care for older people living at home who are acutely ill, rehabilitating, living with a long-term condition or at the end of their life [41, 42]. This wide caseload means that community-based nurses must have a wide range of skills to support the assessment, planning, implementation and evaluation of an older person living with a variety of unique needs at home [37–41]. Further, community nurses are often supported by domiciliary care workers to provide support to people living at home. Domiciliary care, also known as home care, focuses on non-medical or non-nursing support and assistance with daily activities, enabling individuals to maintain independence and quality of life [42].

As highlighted, community nurses, also known as home care nurses, hold a unique position to effectively assess, plan, refer to dental services, and provide support for oral healthcare among older individuals living at home [43, 44]. Moreover, insights from other nursing areas, such as long-term care, reveal that oral care is often deprioritised due to time constraints, inadequate staffing and poor knowledge, leading to potential neglect in this crucial aspect of care [14, 15, 30–36]. Despite this, the authors are not aware of any studies that have explored the experiences of community nurses in the delivery and support of oral healthcare for older people living at home. Accordingly, this study sought to explore this question using a grounded theory approach.

## Methods

### Design

A grounded theory approach was used for the collection and analysis of data. Due to the paucity of research in this area, a grounded theory approach was adopted to support the development of theory in this area [45–49]. This is because findings grounded in the data are more likely to enhance understanding of the current phenomena and present theory that can be used for future action [45–49]. A grounded theory approach also had practical benefits as it enabled the research team to retain a broad focus for this investigation about community nurse experiences in the delivery and support of oral healthcare to this population.

### Sample

The process of recruitment and data collection was guided by theoretical sampling [50]. Theoretical sampling is a form of sampling that is common in grounded theory, and it enabled the research team to seek additional data based on concepts that emerged from the data [50, 51]. In this study, theoretical sampling occurred during the process of data analysis and each new participant was identified because of their role in exploring the research question. This enabled the research team to collect data on community nurses with different levels of clinical expertise, years’ experience and involvement in leadership roles. This approach to sampling was important given the variety of community nursing roles that exist within the current National Health Service in the United Kingdom [52]. Table 1 provides an overview of all study participants.

**Table 1:** Participant characteristics

Pseudonym	Grade of Nurse (Higher Number Indicates Higher Grade)	Gender	Community Area
Abigail	6	Female	Acute Care at Home
Brooklyn	5	Female	District Nursing
Cayleigh	5	Female	District Nursing
Dani	7	Female	District Nursing
Edward	7	Male	Community Mental Health
Faith	5	Female	District Nursing
Georgina	8	Female	District Nursing
Harriet	5	Female	Acute Care at Home
Isaac	6	Male	Community Mental Health
Jessica	6	Female	District Nursing
Khalid	6	Male	Community Mental Health
Lesley	6	Female	Community Learning Disability
Maria	8	Female	District Nursing
Norma	7	Female	District Nursing
Olivia	5	Female	District Nursing

### Recruitment

Study participants were recruited via professional community nursing networks in the United Kingdom. This included the Queen’s Nursing Institute, who are a UK charity and network for community nurses and the Royal College of Nursing, who are the largest trade union for nursing in the United Kingdom. Both organisations circulated a recruitment call seeking expressions of interest for participation in an online semi-structured interview with the research team about the topic area. To be eligible participants first had to be registered nurse in the United Kingdom with active registration on the Nursing and Midwifery Council [53] (NMC) register. They were also required to be currently employed in the role of a community nurse and actively providing nursing care to older people (aged 60 +) in their own home. Interested participants contacted the research team directly to receive further information about the study and arrange a suitable time for interview. Written consent was obtained via an online Microsoft form at least 48 h before the scheduled interview and rechecked again immediately prior to the interview. Fourteen eligible participants contacted the research team for information about the study, ten agreed to be interviewed and four did not respond to the research team after the study information was provided.

In relation to theoretical sampling, snowball sampling was also used to support recruitment of an additional five community nursing participants who could provide valuable insight into the study question. This included one acute care at home team nurse, two community mental health nurses, one community learning disability nurse and one senior nursing leader from district nursing (band 8 grade). All these participants were responsible for initiating contact with the research team after learning about the study from a previous participant. A diverse sample of nurses was essential for this study to capture a comprehensive understanding of the experiences and perspectives related to oral healthcare in home settings. By including nurses from various roles and grades, such as the band 8 senior nursing leader who may not directly perform oral care but is more likely to hold a managerial role, the study can explore the broader implications for leadership and organisational priorities. This approach allows for a more nuanced analysis of the factors influencing oral care provision and potential variations in attitudes and priorities across different nursing levels, contributing to a richer and more well-rounded exploration of the topic.

### Data collection

A total of fifteen community nurses participated in one-to-one semi-structured interviews with one member of the research team (GM). All participants were interviewed online using the Microsoft Teams platform. The interview guide was developed in consultation with all members of the research team, four patient representatives, two representatives from the Queen’s Nursing Institute and four representatives from the Royal College of Nursing. Collectively, this represents expertise from people with lived experience, clinicians from dentistry, clinicians from nursing and colleagues with methodological/research training. The interview guide was refined and guided by theoretical sampling [50, 51]. This meant that questions were added and modified as the interviews progressed, and this is a key aspect of grounded theory methodology. For example, the question *“What are your experiences and challenges in coordinating oral healthcare with other healthcare professionals, such as community dentists or auxiliary nursing staff?"* was added after interview 4 to explore the interprofessional collaboration and communication aspects that emerged during the interviews. As the research gathered more data and insights from participants, it was identified that there was a need to delve deeper into how community nurses work with other professionals to provide comprehensive oral healthcare to older people living at home.

The final version can be viewed in supplementary file 1. Data were collected between May 2021 and December 2021 with interviews lasting between 20 to 50 min.

### Ethical considerations

This study received ethical approval by the Faculty of Health and Life Sciences at Queen’s University Belfast in March 2021 (Ref: MHLS21_29). Written consent was obtained from all fifteen community nurses who participated in this study. All methods were performed in accordance with the Declaration of Helsinki.

In addition to ethical approval and obtaining written consent, rigorous measures were taken to ensure the privacy and confidentiality of the study participants. Personal identifiers were removed during the data anonymisation process to protect the nurses' identities. All data and interview transcripts were securely stored and accessible only to the research team. Any direct quotations used in the study were carefully anonymised to prevent the identification of individual participants or patients. These steps were taken to uphold the principles of data privacy and confidentiality in accordance with the Declaration of Helsinki and other relevant ethical guidelines.

### Data analysis

Data were analysed using constant comparative analysis [50]. Data was therefore collected and analysed simultaneously at the end of each interview before proceeding to the next interview. This approach is common in grounded theory methodology as it enables the findings of each interview to inform the direction for the subsequent interview [46–48]. Following verbatim transcription, data was initially coded line by line. This process of ‘open coding’ supported the research team to identify initial concepts and key phrases in the initial data. The second stage of coding, termed ‘axial coding’, was undertaken to identify relationships between the several categories identified during the open coding stage. Finally, ‘selective coding’, was undertaken to identify one core category which related to all other categories [46–50]. All analyses were undertaken by two members of the research team with significant experience of qualitative analysis in grounded theory research (GM & PS) with input when needed from the wider research team.

### Rigour

The research team employed various strategies to enhance the credibility and trustworthiness of the study’s findings. Initially, when categories began to emerge from the data these were shared with three study participants to ensure they accurately reflected their own experiences, and the emerging theory was applicable. This approach, termed ‘member checking’, provided assurance to the research team that the theory was grounded in the data. As indicated by theoretical sampling processes, the team performed data source triangulation through conduction of in-depth interviews with community nurses of different gender, age, band and clinical speciality. While the inclusion of community nurses with diverse backgrounds may impact traditional data saturation, it enriches the qualitative analysis by capturing a comprehensive range of perspectives on oral healthcare provision for older people living at home. Throughout the study, the research team also ensured rigour through regular team meetings and active involvement of all members as evidenced by a robust audit trail. The study also adhered to the Standards for Reporting Qualitative Research (SRQR) as recommended by O’Brien [54].

## Results

Four categories emerged from the data to support development of the core phenomena. These four categories were: (1) Education, (2) Practice, (3) Confidence and (4) Motivation. The core category in this study, which was apparent across the categories, was defined as (C) Uncertainty. The core category ‘uncertainty’ encapsulated all other categories and emerged to explain how participants in this study managed (1) uncertainty in relation to their education about oral health care, (2) uncertainty in their practice through delivery of good oral health assessment and care, (3) uncertainty about their confidence in supporting patients with a variety of oral healthcare needs and (4) uncertainty about their motivation to optimise their oral healthcare delivery in the future.

### Category 1: education

The first category to emerge from the data was about nurses’ education in relation to oral health. Thinking about their first experiences of oral health education, participants agreed that, while undertaking their pre-registration nursing programmes at different UK institutions, they perceived their taught education in relation to oral health to be satisfactory. Participants unanimously agreed that they developed practical skills in delivery of basic mouth care through a range of formative assessments, including simulated practice, OSCE (Objective Structured Clinical Examination) and through their practice portfolio. While these perceptions were initially positive, the majority of those interviewed went on to discuss how these skills were not built upon in subsequent years.

“I remember that we learned about mouthcare, we had an OSCE [exam] on it and had to get it signed off [in the clinical portfolio] or whatever…but that was probably it. It was a fundamental skill in year one and to be honest, I don’t think we ever went back to it” [Harriet, Acute Care at Home Nurse, Band 5].

The provision of oral health education post-registration was something that all participants felt could be improved. Continuing professional development in the form of postgraduate university modules or programmes, or in-house training led by the National Health Service (NHS UK), did not appear to provide many opportunities for participants to revisit their skills that were initially developed in their pre-registration training.

“I have been working in the community for almost ten years now. I did my specialist practice course in district nursing and to be honest, all things considered, oral health is not something that had a lot of attention. Personally, I don’t neglect this [oral health] – I think you just need to be responsible for developing your own skills in this area” [Maria, District Nurse, Band 8].

While continuing professional development about oral health care appeared to be limited for community nurses, some participants did comment about how they had developed their knowledge about mouth care, particularly when caring for patients at the end of their lives. Provision of palliative care was identified by community nurses as a key part of their role. With regards to supporting patients at the end of their lives to remain comfortable at home, many community nurses had received education about optimising provision of palliative care. This education was often broad, covering aspects such as advance care planning, breaking bad news, sensitive conversations, pain management and spirituality. Providing mouth care to patients at the end of their lives, and teaching family members how to do this, was also included in these types of education.

“We did a course with Macmillan [large UK Cancer charity] and it talked about mouth care. It wasn’t just about that [mouth care] – but a few of the girls [community nurses] did that course and it was useful” [Dani, District Nurse, Band 7].

“So, nothing specifically about oral health [in relation to education programmes], but we do cover it in different courses – maybe not explicitly, but…maybe, so…palliative care training – it is covered then, you know like making someone comfortable at the end and we also do it in dementia training. So, we do cover oral health, but not in its own individual right, no” [Faith, Community Nurse, Band 5].

Study participants shared agreement that, because of the current delivery of oral healthcare education, many did not return to their education about oral health since the first year of their pre-registration programme. While some community nurses were not concerned about this gap in education, most felt concerned that they could have gaps in their knowledge about new and improved practice. Furthermore, few community nurses were aware of where they could go or who they would talk to about receiving updated training about oral health care in relation to their role as a community nurse.

“As you’re asking me all these questions today [directed to interviewer], it occurred to me quite quickly that I probably don’t know enough [about oral health]. It [oral health] just isn’t a big priority I suppose…we are doing updates [training] all the time about things like syringe drivers [battery-powered pump that delivers medication intramuscularly] that are vital for patient safety you know? But when it comes to something like mouth care – has practice really changed that much? A mouth is still a mouth. I am not sure, but you’ll probably say to me that a lot has changed, and I am completely ignorant to this because I haven’t updated my knowledge!” [Jessica, Community Nurse, Band 6].

Overall, this category has articulated that continuing professional education opportunities about oral healthcare is limited amongst community nurses. While some community nurses may receive updates to their knowledge, this will likely come on an ad-hoc basis or through coverage on another course. As a result, many community nurses are required to rely on oral health knowledge attained through their pre-registration training.

### Category 2: practice

A second important category identified from the analysis related to oral health in the assessment, support and practice in the context of nursing practice. Participants identified limitations in current delivery of oral healthcare to their patients across all four of the main aspects of the nursing process; (1) assessment of oral health, (2) planning oral health care interventions, (3) implementation of planned oral health interventions and (4) evaluation of oral health care practice [55].

Considering assessment, all community nurses interviewed stated that oral healthcare assessment was not routinely completed as part of their practice. The main reason for this was because there appeared to be no standardised assessment document for community nurses to complete as part of their visit. Another reason for this was due to the nature of community nursing practice, namely community nurses may only see patients for a very short time and provide very specific care that may not necessitate an oral health assessment, for example administration of insulin or wound care were the most cited examples.

“It isn’t something that is probably looked at (oral health) especially for a one-off (monthly visit), bar just asking how the patient is and if they say, ‘I have a sore tooth’ or something, we probably wouldn’t look at their mouth…I suppose we could neglect that area. [Edward, Community Mental Health Nurse, Band 7].

While there was no standardised assessment document about oral health across participants, many community nurses referenced various NHS assessment tools that they used for care of long-term care patients. These often-included brief consideration of oral health as noted here.

“We use the NISAT (Northern Ireland Single Assessment Tool), you know it? It asks about the condition of the mouth. So, on initial assessment, it would be asked, and it would be checked…we initially check the NISAT when we are bringing someone onto the caseload. [Brooklyn, Community Nurse, Band 5].

On examination of the NISAT [56], the authors noted two questions about oral health of patients. These included ‘have you any concerns about your mouth or teeth’ and ‘what affects your ability to eat and drink’. The full version of these assessment questions is noted on Table 2.

**Table 2:** Oral health assessment in the community example questions

Have you any concerns about your mouth or teeth?
Please consider:
• Painful teeth or gum
• Bleeding gums
• Ulcers, lumps or bumps
• Difficulty speaking
• Dry mouth
What affects your ability to eat and drink?
Please consider:
• Illness
• Quality/amount
• Stress/worry
• Painful mouth/gums
• Dentures
• Appetite
• Likes/dislikes/choice
• Availability of Food
• Other

Overall, participants agreed that oral health assessment was not standardised or routine. While there was some evidence of good practice, oral health assessment was often prompted by patients who had mouth pain. Implementing good oral health practice was often the role of domiciliary or home care workers. These domiciliary care workers would often visit the older person daily and enable them to remain independent at home by helping with their personal care (including mouth care), medication, pet care, meal preparation and cleaning [57]. Participants agreed that their relationship with domiciliary care workers was important, but also that delivering oral health was an element of personal care that was often beyond the remit of community nurses.

“Definitely my team (community nursing) would take the lead in patient assessment and care planning – 100%. My team would then share that plan with dom-care (domiciliary and home care workers), who would go on to implement it…because mouthcare falls under ‘personal care’, that wouldn’t be within our daily role strictly” [Norma, District Nurse, Band 7].

“I am not sure if I would say we work closely [community nursing and home care workers]. We certainly work in partnership, but I couldn’t tell you the name of the person [home care worker] who is going into the patient’s home or what exactly they do…but I do know, mouth care and personal care – doing it, is their remit” [Cayleigh, Community Nurse, Band 5].

Community nursing practice, as it related to supporting assessment, planning and implementation of oral health was well described by participants. While there were clear areas for development, all participants could talk to aspects of these in their role. An area that was less well described amongst community nurses, related to their role in providing preventative advice to older people living at home. Most community nurses interviewed discussed how they had provided some information about oral cleanliness, lip care, gum and tissue care and denture care in their role, however this appeared to be on an ad-hoc basis and was patient specific. Only two senior community nurses discussed how they, or their teams, had roles in providing advice in relation to modifiable lifestyle factors that could promote good oral health.

“As community nurses we could provide [lifestyle] advice to patients about smoking, alcohol, high sugar diets. Of course, we are a very busy service, so this is not always possible because of the short time we have per visit.” [Jessica, District Nurse, Band 6].

“No, I think this [in relation to providing advice about modifiable risk factors] is a bit aspirational. I am sure our girls and boys [community nurses] are aware of the information [modifiable lifestyle factors], but really, on the front line we are sometimes struggling to meet the basic safety needs of patients” [Georgina, District Nurse, Band 8].

Community nurses have a significant role in the assessment, planning and implementation of oral health care to older people living at home. Practices amongst community nurses appeared varied. Additionally, there were no participants that discussed how oral health practice was evaluated by community nurses (after subsequent discussion on assessment, planning and implementation). Furthermore, it was evident that community nurses did not routinely provide direct oral health care to patients and, due to time constraints, had a limited role in supporting older adults in health and oral health promotion.

### Category 3: confidence

The third key category to emerge from data analysis was confidence. This category had significant overlap with category one (education) and category two (practice) because both had strong influences on community nurse confidence in oral health care. For example, in the coding of analysis, it was noted that low levels of education often led to low levels of confidence, and inconsistent practices were likely to be because of this low confidence. Despite this overlap, the category of confidence was evident in analysis and therefore is warranted as a standalone category.

Community nurses had variable experiences in relation to partnership working with oral health professionals. Of the fifteen participants interviewed, only five had experience of liaising with community dentists. In the UK, community dentists are dental professionals who provide oral healthcare services primarily outside of private practice settings, often working in community clinics, schools, or other community-based settings. All five of these participants, who practised in different geographical areas, noted that the motivation for contacting the community dentist was due to either dental pain or an issue with a patient’s dentures and reported that community dental services were effective in supporting the patient after involvement.

“It was quite straightforward really, the patient’s dentures didn’t fit right and kept falling out. They didn’t have a dentist, so when I got back to the office [after seeing the patient in their own home], I arranged the referral.” [Isaac, Community Mental Health Nurse, Band 6].

“Yes, I would have worked with the dentists a little bit, they wouldn’t be the professional we speak with most by any means though…In the beginning, I wasn’t sure who to speak to about getting a community dentist, but after some digging [searching for answers], I got a contact number, and it was done. I keep this contact information as it still comes up now and again [patient needing referred]”. [Dani, District Nurse, Band 7].

While there was some evidence of collaborative working, most community nurses that were interviewed were not sure about how to contact an oral health professional if the need arose. Furthermore, except for one community nurse, all participants stated that they did not routinely ask patients about the date and outcome of their last dental visit and therefore did not consider how they could support their patients to find a new dentist if needed.

“I wouldn’t know where to start to be honest [in helping a patient find a dentist]. Our time is extremely limited, and we wouldn’t really have scope to sit down with the yellow pages [telephone directory in the UK] and do this” [Norma, District Nurse, Band 7].

“It wouldn’t be part of our job to ask about dental visits. I think that is probably a bit unrealistic in all honesty because where would you draw the line? I know teeth are important, but so is sight, hearing and so on. Do you know what I mean? Honestly, we’d be there all-day doing assessments and arranging things.” [Jessica, Community Nurse, Band 6].

Throughout semi-structured interviews, community nurses articulated how they delivered oral healthcare to their patients who lived at home. As a result, it was determined that participants had high confidence in providing oral healthcare that focused on patient’s oral mucosa. Oral mucosa, the lining inside of the mouth, was of key importance to patients at the end-of-life and most participants highlighted how they delivered high quality mouth care to their patients.

“Mouth care at end-of-life is our bread and butter [a fundamental part of the job] to be honest. Yes, there is room for improvement, but I think our team is good at this.” [Georgina, District Nurse, Band 8].

“It is a big part of the job, and I am quite comfortable in doing this [mouth care at end of life] and showing the family how to do this too. For us it is about keeping the mouth clean, moist and comfortable.” [Olivia, District Nurse, Band 5].

Participants also discussed how they felt confident in providing care to other aspects of a patient’s oral mucosa including their lips and tongue. This confidence in delivery of oral health care did diminish when consideration was given to their role in caring for patient’s teeth. Many participants in this study expressed that they saw their role as keeping the mouth comfortable, clean and moist. While acknowledging that good toothcare was a vital part of a person’s overall health, few appeared to have confidence in this area.

“I am not even sure how many teeth a person is meant to have to tell you the God’s honest truth”. [Cayleigh, District Nurse, Band 5].

“That is the job for the dentist surely [teeth]. I wouldn’t know where to start when assessing someone’s teeth”. [Khalid, Community Mental Health Nurse, Band 6].

“To me they are two different things – mouth care is more about the mouth tissues, and we can do that quite well [nurses], but teeth are best left to dentists I think”. [Jessica, Community Nurse, Band 6].

Following on from this, and with consideration to providing health promotion advice about oral health care, few community nurses discussed modifiable lifestyle factors with their patients as noted in category two. While time constraints have been postulated as one of the key factors for this, some participant interviews also illustrated that a key factor may be related to lack of confidence in this area.

“In an ideal world, where the workload demands are not as intense, it would be great to have that discussion with patients [about modifiable lifestyle factors]. Of course, it would. But we’d all probably need a refresher course or some training about how to deliver it though”. [Edward, Community Mental Health, Band 7].

“You mean like sugary diets or erm…like not brushing or teeth [in reference to modifiable risk factor discussion with patients]. Yes, I suppose I might do this now and again, just the basics – eat five fruit and vegetables and take vitamins or whatever. But it isn’t a normal part of the job for me.” [Abigail, Acute Care at Home Nurse, Band 6].

Overall, it was apparent that community nurses appear to have confidence in providing good mouth care to their patients, particularly at the end of their life. The main areas where community nurses seemed to lack confidence related to care of patient’s teeth, how to engage dental services in their role if required and how to provide health promotion to patients about their oral health.

### Category 4: motivation

The final category that was generated from data analysis related to motivation. This category presented the greatest variance in responses amongst participants with some community nurses stating they were keen to develop their delivery and support related to the oral health care to patients, while others felt that the role of a community nurse was already too busy.

The desire for Community Dental Services to take more of a leadership role in supporting patients’ oral health at home was articulated by about half of the participants interviewed. Community nurses that participated in this study felt that their workload was already too great, and their remit was almost always more than a person’s mouth (for example, end-of-life care, administration of insulin, providing wound care and so on). As such, some participants felt that Community Dental Services should be responsible for patient’s living at home.

“Notwithstanding the whole system is broken, we can’t do everything [community nurses]. There is no slack in the system and we’re already doing too much. The care of someone’s teeth is the job of the dentist and it is up to them to sort this out [provision of better oral health to people at home]”. [Jessica, Community Nurse, Band 6].

“I mentioned the word aspirational a few times in this interview and I am sorry for that because this is important [oral health]. But let’s be realistic – we’re overworked, underpaid and demotivated. Of course, we want the best for our patients, but the NHS is meant to be about teamwork and sometimes I feel we are doing it all”. [Georgina, District Nurse, Band 8].

“We all have our roles, SALT (speech and language service) can help when someone isn’t able to swallow, a TVN (tissue viability nurse) can go out to someone’s home if there is a particularly nasty wound, GPs (general practitioners) can get the person prescriptions, and the dentists look after teeth”. [Brooklyn, District Nurse, Band 5].

On the contrary, there was also evidence that participants in this study felt that they were in an advantageous position of seeing older people at home, often living with complex needs, and therefore community nurses could be the key professional group in terms of patient oral health.

“To be honest, I think there is an exciting opportunity for our service [community nursing] to do a bit more in this area and role-model best practice” [Faith, Community Nurse, Band 5].

“There is a QI [quality improvement] project bubbling around in my head now about all this [community nursing role in oral healthcare]”. [Olivia, Community Nurse, Band 5].

“In theory, yes, we are seeing a high-risk population that are probably not seeing a dentist anymore. So, in a way we are well placed to take a bit more leadership in this area”. [Maria, District Nurse, Band 8].

While category four was less detailed than the previous three categories, it is significant owing to the divergence in opinion amongst participants. Succinctly, in this study there were about half of the community nurses that were keen to develop their provision of oral healthcare, while the other half felt that doing anymore was beyond their role.

### Core category: uncertainty

Identification of a core category is a central feature of grounded theory methodologies [45]. This is the final stage of data analysis and involves the presentation of one core category that includes every part of the data set presented (in this case related to education, practice, confidence and motivation). The core category therefore serves as the foundation for the development of theory [46]. In this case, *‘uncertainty’* was the core category as it recurred frequently in the data, it was at the centre of the study, interacted with all aspects of the study, and is logical because it naturally appeared from the data set [45–50].

Community nurses in this study articulated *uncertainty* about their role in providing oral healthcare to older people living at home. With regards to (1) education, it was illuminated that provision of oral healthcare education was suboptimal and most community nurses were *uncertain* about where they could receive further education. In addition, community nurses were often *uncertain* about whether they required further education on this area because of the existing complexities of their role and the fact the oral health is perceived as a basic nursing skill. These elements appear to oppose one another and manifested with community nursing uncertainty about whether they required continuing education on oral health or whether provision of this education in this area was necessary given the perceived basic nature of the skill.

In reference to (2) practice, there was *uncertainty* around how oral healthcare should be provided in the context of the nursing process. The absence of standardised assessment tools for oral health led some community nurses to feeling *uncertain* about whether they were doing enough in this area. Category two also illustrated that there was *uncertainty* about implementation of routine oral healthcare practice, in this case the differing roles between community nursing and home care workers. Furthermore, category two also demonstrated that community nurses were *uncertain* about their role in providing oral health promoting advice to their patients.

Considering (3) confidence, participants in this study expressed *uncertainty* about working collaboratively with oral health professionals, their role in asking questions about patient’s previous dental visits and how to help older people living at home contact or find a dentist. Throughout category three, it was also found that community nurses were *uncertain* about how best to assess and provide care that affected an older person’s teeth. This *uncertainty* also extended to providing advice to patients about modifiable lifestyle factors such as diet, smoking and hygiene.

The final category (4) was about motivation, and this highlighted that many community nurses interviewed in this study were *uncertain* about their ability and desire to be part of future change in oral healthcare service provision. These participants also appeared to feel *uncertain* about the extent to which oral health was part of their role. Those participants that demonstrated interest and appeared motivated to consider how practice could change also expressed *uncertainty* about how this could best be taken forward.

Overall, *uncertainty* was a feeling that was expressed by all participants across all four presented subcategories in relation to provision of oral healthcare for people living at home. This is also evidenced in the excerpts that follow.

“Honestly, what we’re talking about is a bit of a mine field. Is oral health important, yes. Do we do it well, sometimes. Do we all work together, not always. What is the role of the nurse [community nurse] in all this, I don’t think that is clear. There is a lot of uncertainty in practice”. [Brooklyn, District Nurse, Band 5].

“Before you go [to interviewer before interview concludes], I want to ask you something for my own benefit if I could be so bold…The assessing, planning, delivery and referral of oral health for patients at home, do you think that should be all on us [community nurses] and if it isn’t, whose role is it anyway?” [Edward, Community Mental Health Nurse, Band 7].

## Discussion

Oral health needs and oral health-related quality of life amongst older people receiving care in their own home is an important issue. A recent scoping review by Henni and colleagues mapped the literature on oral health indicators and oral health-related quality of life in older adults receiving home health care services [58]. This review, comprised of 18 empirical studies, indicated that older people who received formal care at home (that is care from community nurses, domiciliary care workers and other support staff) often lacked some of their natural teeth and often had removable dentures that needed repair. The review also reported that plaque, caries, xerostomia, and chewing and swallowing problems were common among this population [58]. In the context of the present study, community nurses are the healthcare professionals that provide care to older people with the most complex needs (for example those living with dementia, heart failure, COPD, frailty and so on). It is therefore logical to postulate that these patients are the most likely to require support with their oral health also. Despite this, there have been a paucity of empirical research studies that have sought to explore the experiences of community nurses in this area.

The core category that has been generated from data analysis has theorised that community nurses are uncertain about their role of oral health delivery and support for patients that live at home. One of the main reasons for this was because most participants learned about oral health when training to become a nurse and few revisited this or received updating education on oral health after qualifying. The lack of continuing professional development activities explicitly linked to oral health may have deprioritised this area of care for community nurses. At a global level, the World Dental Federation (FDI) strongly advocates that improvements in interprofessional education, including community nursing, is important to improve the oral health of patients [59]. A scoping review by Wilson and colleagues around intellectual disability and oral health, the authors noted a key theme of their evidence synthesis was also around uncertainty regarding the efficacy of specific tooth brushing interventions for people with intellectual disability [60]. Such uncertainty of role has also supported by empirical research carried out with non-community nurses. For example, in care homes [61–63] and hospitals [64, 65].

The provision of oral health education has the potential to increase capacity to reach and support vulnerable older people in any care setting [66]. Despite this importance, a recent systematic review, comprising 11 empirical studies about undergraduate nursing curricula, found that nursing students had limited knowledge and varying attitudes about the importance of oral health [24]. A systematic review also reported there was a lack of educational interventions that were available to support nursing student understanding about oral health [24]. These international findings concord with the present study. Education about oral health care is recognised as being very important for community nurses, however there is an uncertainty about how best practice should look owing to the high likelihood of suboptimal education pre and post registration as evidenced by this study and the aforementioned reviews.

This study also found that the absence of good education about oral health appeared to impact on practice and the subsequent delivery and support of patient oral healthcare. In other words, low oral health literacy amongst community nurses is likely to be a key factor in uncertainty around optimum practice. However, it is also important to note that good knowledge will not always translate into better oral healthcare. Indeed, optimum practice in oral health is predicated on good relationships with other professionals, namely domiciliary care workers and community dentists in the case of community nurses. Interprofessional collaboration has been identified as a main facilitator for the integration of good oral health care to older people in primary care [43, 67]. While these professional relationships were also important in the context of the present study, there appeared to be limited collaboration between community nurses, domiciliary care workers and Community Dental Services. This is reflective of the international evidence-base which suggests that many non-dental health professionals may have negative attitudes towards the oral healthcare of their patients [68–70].

Community nurses have a challenging role in practice. In the United Kingdom, a recent cross-sectional survey of more than 3000 registered nurses working in community settings found that approximately 34% of care was ‘left undone’ [71]. The same study also reported that 37% of community nursing respondents reported that they had the planned number of nursing staff on their previous shift [71]. These factors provide important context for the present study in which many community nurses articulated that time constraints were a significant barrier to optimum delivery of oral healthcare and prohibited important discussions from taking place with patients about dental visits or diet modifying behaviour that could sustain and improve oral health.

## Strengths and limitations

Given this lack of research in this important area, a key strength of this study is the use of grounded theory methodology. These methods were used to support the researchers to develop theory in this area. The use of theoretical sampling, and subsequent inclusion of a variety of community nurses with different roles and experiences is also a strength and the resulting theory has potential applicability across the range of roles within community nursing.

A limitation of this study was the sample. As recruitment was supported by the Royal College of Nursing and the Queen’s Nursing Institute, this meant that community nurses who were not part of these networks could not be included. In addition, data collection took place via online semi-structured interviews. While this was a convenient way to engage with participants and was in line with COVID-19 guidance at the time, online interviews may have made it more challenging for the research team to develop a rapport with participants and gain a deep understanding of the research question [72].

While the band of nurse, type of community nurse and gender of participants were collected during the study, a limitation to this was the absence of data on how long participants had been qualified as a registered nurse and how long they had worked as a community nurse. These demographics may have provided further insight into study findings.

## Conclusion

In conclusion, this study shed light on the uncertainty experienced by community nurses in providing oral healthcare to older people living at home. The findings underscore the need for comprehensive and ongoing education in oral health for community nurses to enhance their confidence and efficacy in supporting patients. Interprofessional collaboration and clearer delineation of roles with oral health professionals could also help address uncertainty and improve oral health outcomes for this vulnerable population. Ultimately, understanding and addressing this uncertainty will be essential in developing effective strategies to improve oral health care for older adults in community settings.

### Supplementary Information

**Additional file 1.**

## Data Availability

The datasets used and/or analysed during the current study are available from the corresponding author on reasonable request.
