# Supplementary material for: ‘Whose role is it anyway?’ Experiences of community nurses in the delivery and support of oral health care for older people living at home: a grounded theory study
Source: BMC Nurs. 2023 Oct 5;22:359. doi: 10.1186/s12912-023-01533-0 (PMC10557176; doi:10.1186/s12912-023-01533-0)
Supplement: Supplementary file 1 — Additional file 1. [file 12912_2023_1533_MOESM1_ESM.docx]

**Supplementary File 1: Study Interview Guide**

Community nursing healthcare provision

- What role to you have in relation to providing or supporting the oral healthcare of older patients living at home?
- To what extent do patients have their oral health regularly assessed by community/district nurses?
- Is there a particular profile of patient that requires higher levels of oral care in the community (e.g. people with dementia, people living with advanced cancer).
- Other than community/district nurses, what other people have a key role in the provision of oral care to these patients (e.g., family members, auxiliary nursing staff, community dentists).
- How much input does your community/district nursing service have with colleagues from dentistry (e.g., community dentists)?
- What sort of education or training is provided for community/district nurses about oral healthcare of older patients and what are your thoughts on this? (e.g., is it mandatory training, does it meet your professional needs?)

Views and perceptions of oral healthcare service older people living in the community

- How important do you feel it is for community/district nurses to be involved in the oral care of patient’s living in the community?
- What do you believe are the main barriers for community/district nurses in providing oral healthcare to older patient’s living at home? (e.g., COVID-19, financial and time constraints, lack of equipment, lack of knowledge, safety, complex needs etc.)
- What helps you as a community/district nurse to ensure optimum practice in relation to providing oral healthcare to your patients.
- How do you think we can improve provision of oral healthcare for older people living in community settings?

*Conclusion:*

- Do you have anything else you would like to add that we haven’t already discussed?
- Thank you for your contribution.
- Ask the participant if there are any statements that they do not wish to be transcribed.
